# Pathologic Blood Samples Tolerate Exposure to Vibration and High Turbulence in Simulated Drone Flights, but Plasma Samples Should be Centrifuged After Flight

**DOI:** 10.1109/JTEHM.2021.3053172

**Published:** 2021-01-20

**Authors:** Karl Arne Johannessen, Nina Kristin Steen Wear, Karin Toska, Morten Hansbø, Jens Petter Berg, Erik Fosse

**Affiliations:** 1 The Intervention CenterOslo University Hospital 155272 0188OsloNorway; 2 Faculty of MedicineInstitute of Health and Society, University of Oslo 6305 0372OsloNorway; 3 Department of Medical BiochemistryUniversity of Oslo 6305 0315OsloNorway; 4 Department of Medical BiochemistryOslo University Hospital 155272 0188OsloNorway; 5 Faculty of MedicineInstitute of Clinical Medicine, University of Oslo 6305 0372OsloNorway; 6 Faculty of MedicineInstitute of Basic Medical Sciences, University of Oslo 6305 0372OsloNorway; 7 The Norwegian Defense Research Establishment2007OsloNorway

**Keywords:** Biologic tolerance, drone transport, health care

## Abstract

Objective. Most of the previous studies of drone transport of blood samples examined normal blood samples transported under tranquil air conditions. We studied the effects of 1- and 2-hour drone flights using random vibration and turbulence simulation (10-30 g-force) on blood samples from 16 healthy volunteers and 74 patients with varying diseased. Methods: Thirty-two of the most common analytes were tested. For biochemical analytes, we used plasma collected in lithium heparin tubes with and without separator gel. Gel samples were analyzed for the effect of separation by centrifugation before or after turbulence. Turbulence was simulated in an LDS V8900 high-force shaker using random vibration (range, 5–200 Hz), with samples randomly allocated to 1- or 2-hour flights with 25 or 50 episodes of turbulence from 10 to 30 G. Results: For all hematologic and most biochemical analytes, test results before and after turbulence exposure were similar (bias < 12%, intercepts < 10%). However, aspartate aminotransferase, folate, lactate dehydrogenase and lipid index increased significantly in samples separated by gel and centrifugation prior to vibration and turbulence test. These changes increased form 10 G to 30 G, but were not observed when the samples were separated after vibration and turbulence. Conclusions: Whole blood showed little vulnerability to turbulence, whereas plasma samples separated from blood cells by gel may be significantly influenced by turbulence when separated by spinning before the exposure. Centrifugation of plasma samples collected in tubes with separator gel should be avoided before drone flights that could be subject to turbulence.

## I. Introduction

Unmanned aerial vehicles (UAVs, hereafter termed drones), have been tested for a broad range of civil applications, including industrial surveillance, business parcel delivery, and imaging. In health care alone, search-and-rescue of survivors of natural disasters, delivery of medicines and vaccines to rural areas and care technology devices to emergency situations, and rapid transport of blood samples and organs, are some of the possible drone uses that have been investigated [1]–[8]. However, if drones are to provide such eruptive changes to the transport of biologic materials, they must offer near-100% uptime with acceptable quality of biologic specimens. To assess whether drone transport represents a sustainable alternative to the existing high-schedule transport systems, we need real-life situations to identify and overcome potential problems that may arise under challenging conditions, such as wide ranges in wind, precipitation and temperature, turbulence around and across infrastructures (i.e. around buildings and varying terrains) and more.

Maintaining the quality of biologic samples during aerial transportation is an important prerequisite for the regular use of drones in critical health transport services. Most undesired vibrations and effects of severe weather-induced turbulence can be dampened technologically, but not all; certain vibrational frequencies are poorly dampened and vigorous turbulence may not be fully absorbed.

Until now, most published studies on the transport of biological materials have used blood samples from healthy volunteers and were conducted under favorable meteorological conditions (temperature, wind, and precipitation) [1], [7], [9]. Although the studies by Amukele *et al.* [1] demonstrated the proof of concept with respect to both distance and time flown, their studies attempted to diminish the effects of vibration and flight turbulence. They also pointed out that we do not know the impact of drone transport on blood analytes that are outside normal reference ranges, or the effect of flights exposed to more challenging environmental conditions with respect to temperature and wind.

Transport in locations such as Norway will require drone performance under demanding weather conditions that may create turbulence and other physical influences, despite the best available technical precautions. Accordingly, we must address these performance gaps before drones can be realistically considered for large-scale applications. The effects of g-forces on blood samples have been studied with regard to centrifugation and validation of pneumatic tube transport [10]–[14], but to the best of our knowledge, no studies have addressed vibration and turbulence during drone transport.

Natural occurring wind conditions are difficult to predict and control, making it challenging to a priori define a range of wind conditions that could be tested to elaborate limits which may critically threaten biologic quality or integrity during actual drone flights. Therefore, we developed a model to perform in vitro vibration and turbulence experiments in a laboratory where we could simulate and quantify a variety of possible physical conditions of extreme situations on drone flights.

In the present study, we simulated 1- and 2-hour drone flights using an extensive range of vibrations and turbulence with g-forces ranging from 10 G to 30 G to test the effects on blood samples from patients with a wide range of abnormal blood values.

### A. Research Questions

Oslo University Hospital is located in an area that experiences extreme, varying meteorological conditions, including temperatures that range from −30°C to +30°C and excessive wind; therefore, we considered the effects of turbulence related to such conditions on drones to be a crucial study topic.

Our main goal was to study the effect of turbulence and vibration exposure on different biochemical substances in a range that reasonably replicates the conditions for drone flights in our geographic region. We experimented with turbulence levels ranging from 10 to 30 G, and 1 and 2 hours of exposure to evaluate the effect of flight time. These exposure times were chosen because they covered the aspects of our two ongoing projects: 1) a time-intensive, high-volume transport project for consolidation of large laboratories involving < 1-hour flight distance; and 2) a 2-hour flight project in another hospital with less activity and lower need for time intensive transport volumes, but with transport distances of up to 200 km Euclidian distance. We considered 30 G as a maximum force relevant for drone transport because it covers a speed change from 100 km/hour to zero in 0.1 sec. We did not consider it relevant to test complete crash simulation in this study.

## II. Methods

### A. Blood Samples

On test days, surplus blood samples drawn from patients between 04 and 09 AM were collected in the Departments of Medical Biochemistry at the Ullevål and Rikshospitalet of Oslo University Hospital in Oslo, Norway. The samples were randomly collected, but we ensured that we included patients from our clinics with malignant diseases, leukemia, hematologic and infectious diseases, and other routine patients in our inpatient and outpatient clinics. To ensure that the sample materials contained both normal and pathological values, we also included blood donations from 16 normal volunteers who provided informed consent.

Morning collections were chosen to facilitate recruitment of patients receiving tests in the fasting state and to enable us to conduct turbulence testing and return to the laboratory within normal working hours. All blood samples were drawn from inpatients and outpatients using standard routine procedures. Vacuette collection tubes from Greiner Bio-One were used as follows: tubes coated with anticoagulant K2 EDTA for hematologic analytes; tubes containing sodium citrate were used for coagulation analytes; and lithium heparin tubes with and without separator gel were used for biochemical analytes. All analytes were measured both before and after turbulence testing.

**FIGURE 1.**
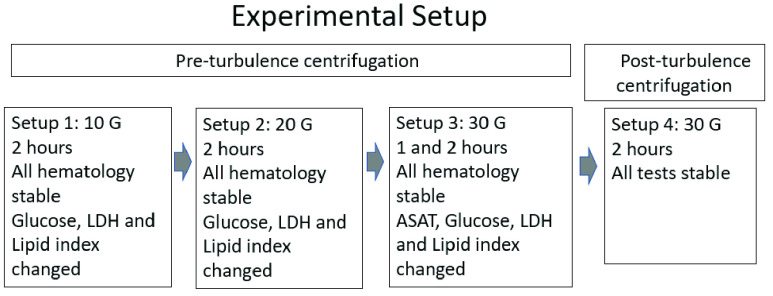
Setups 1–3 used pre-turbulence centrifugation with an identical centrifugation method for control and test samples, with g- force increasing from 10 to 30 G. Setup 4 used post-turbulence centrifugation at 30 G for all samples.

Our experimental setup is depicted in Figure 1. We had no a priori knowledge of which turbulence levels would be relevant to test. To identify g-levels that may damage blood samples, and based on studies of g-forces in centrifugation and PTS transport systems, we started at 10 and 20 G for 2 hours, assuming lower g-levels would be too modest. As most samples appeared to be stable, the test was increased to 30 G and applied over 1- and 2-hour periods in order to determine if higher g-levels cause more damage to analytes. However, even at maximum turbulence, there were no additional changes in analytes above those observed at lower g-levels.

In setups 1–3, control and test tubes containing separator gel were spun by centrifugation before turbulence was applied with a Roche Cobas 8100 automated workflow series at 4,150 rpm (3,$004\times $ g) for 5 min. In setup 4, control and test tubes, both with and without separator gel, were spun by centrifugation after turbulence with a Kubota s700 TR at 3,410 rpm (2,$500\times $ g) for 10 min. Control samples were stored in the laboratory, and all samples were kept at room temperature (18–22°C) at all times. All test samples were spun on the same system and at the same time as their corresponding control.

The samples were randomly allocated to 1- and 2-hour flights without knowledge of the patient’s status; no padding was used. After completion of the simulations, samples were transported to the laboratory where control and test samples then were analyzed. The mean time span between patient blood retrieval and the completion of turbulence simulation was 5.8 hours (range, 5.2-8.8 hours, standard deviation 0.98 hours).

**FIGURE 2.**
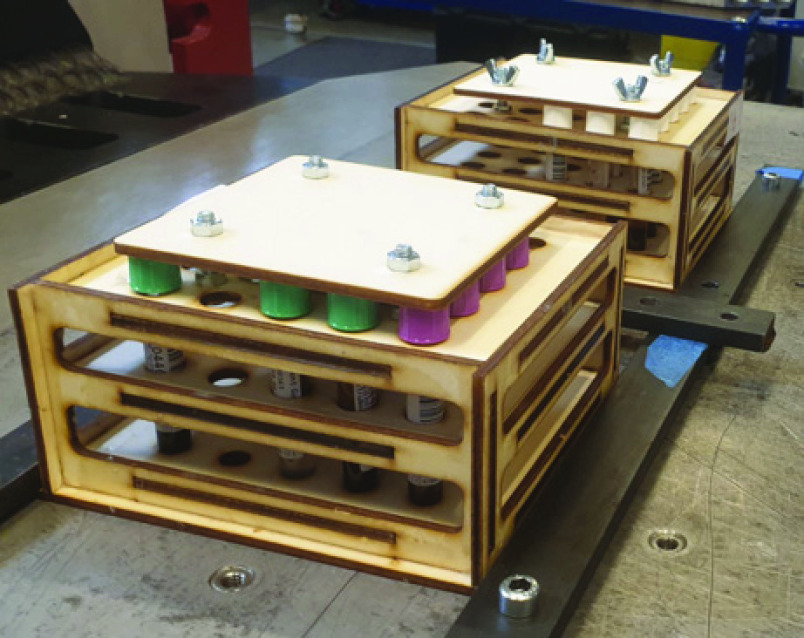
3-D printed plywood boxes for transport and testing.

The test samples were transported by car (20 minutes’ drive), held in vertical position in special 3D-printed carbon plywood boxes adapted with a close-to-perfect fit for the tubes to minimize shaking within the box (Figure 2).

We tested 32 of the most frequent analytes in our laboratory (Table 1 and 2). The hematologic analytes were analyzed on a Sysmex XN-9000 (Sysmex Corporation, Kobe, Japan), the coagulation analytes on a Stago Sta-R (Stago, Asnières sur seine, France), and the biochemical analytes on a Roche Cobas 8000 (Roche Diagnostics, Risch-Rotkreuz, Switzerland). All control and test samples were analyzed on the same system.

**TABLE 1:** Mean Values of the Hematologic, Biochemical and Coagulation Analytes in Control and After Test in the Analytes that Did Not Change After Exposure to Turbulence. (P-Values for All = Nonsignificant)

	Analysis	Normal Value	RCV (%)	Control Mean	Control Minimu m	Control Maximum	Control SD	Test Mean	Test Minim $\mu {\mathrm{ m}}$	Test Maxim $\mu {\mathrm{ m}}$	Test SD	N	% Mean Change after Turbulence
Hematology	Basophiles	0 -0,1*10^9/L	11,3	0,0	–	0,2	0,0	0,0	–	0,2	0,0	59	−5,2 %
	Eosinophiles	0 -0,4*10^9/L	20,2	0,1	–	1,2	0,2	0,2	–	1,3	0,2	59	3,6 %
	Hematochrit	0,35-0,50	0,1	0,4	0,2	0,5	0,1	0,4	0,2	0,5	0,1	59	−0,1 %
	Hemoglobine	11,7-15,3 g/dL	9,8	11,7	6,5	16,3	2,8	11,6	6,5	16,2	2,8	40	−0,6%
	Lymphocytes	1,1-3,3*10^9/L	35,8	1,4	0,0	5,2	1,0	1,4	–	5,2	1,0	59	−1,1 %
	Mean corpuscular hemoglobin	27-33 pg	7,3	30,5	19,8	36,4	2,8	30,5	19,9	37,5	3,0	59	0,2 %
	Mean corpuscular hemoglobin concentration	31-35 g/l00 mL	4,2	32,7	26,6	36,0	2,0	32,7	26,4	36,3	2,1	59	−0,1 %
	Mean corposcular volume	82-98 fL	6,16	93,3	72,9	108,7	7,0	93,6	73,3	108,7	7,2	59	0,4 %
	Monocytes	0,2 - 0,8 + 10^9/L	23,3	0,7	0,0	2,3	0,5	0,7	0,0	2,4	0,5	59	0,2 %
	Mean platelet volume	9,6 13,1 f/l	0,1	10,3	–	12,2	2,0	10,5	–	14,5	2,8	40	1,2 %
	Neutrophiles	1,5 - 7,3*10^9/L	9,4	4,5	0,0	20,3	3,9	4,5	0,0	20,2	3,9	59	−0,6%
	Platelet count	145-390*109/L	8,5	192,4	2,0	991,0	206,3	197,0	2,0	989,0	204,4	40	2,4 %
	Red blood cell count	4,3-5,7 *10^12/L	4,8	3,8	2,3	5,2	0,9	3,8	2,3	5,2	0,9	59	−0,4%
	Reticulocytes	30 - 100 $\times$ 10^9/L	13,2	26,4	–	146,0	40,1	23,9	0,1	131,0	35,7	59	−9,6%
	White blood cell count	3,5-10,0*10^9/L	9,6	6,7	0,0	23,5	4,5	6,7	0,0	23,5	4,5	59	−0,6%
Biochemistry	Alat	10-70 U/L	26	104,6	6,0	3 119	435,4	104,3	6,0	3 013	420,2	51	0%
	Calcium	2,15-2,51	4,5	2,2	1,9	2,6	0,2	2,2	1,9	2,6	0,2	31	2 %
	Chloride	98 -107 mmol/L	2,9	101,4	84,0	126,0	6,5	101,2	85,0	125,0	6,3	31	0%
	Crp	<4 mg/L	42,9	94,8	4,3	321,1	101,9	93,9	4,3	320,4	100,4	31	−1 %
	Magnesium	0,71-0,94 mmol/L	5	0,8	0,7	1,1	0,1	0,9	0,7	1,1	0,1	31	4 %
	Potassium	3,5-4,4 mmol/L	5,1	4,2	3,1	5,3	0,5	4,3	3,3	5,4	0,5	31	3 %
	Sodium	137-144 mmol/L	1,6	139,4	131,0	163,0	6,3	139,3	131,0	163,0	6,4	31	0%
	Triglyceride	0,5-2,6 mmol/L	20	1,9	0,3	4,6	1,0	1,8	0,2	4,4	1,0	20	3 %
Coagulatior	Activated Partial Thromboplastin Time	<30	11,2	33,5	30,0	40,0	3,1	33,6	30,0	40,0	2,8	20	0%
	D Dimer test	<0.3	66,9	0,9	0,3	4,0	1,1	0,9	0,3	4,0	1,1	30	1 %
	Fibrinogen	2.0	42,3	3,8	1,2	8,0	2,0	3,7	1,2	8,1	1,9	30	−1 %
	INR test	<1.1	7,0	1,1	0,9	1,9	0,2	1,2	0,9	1,8	0,2	30	2 %

**TABLE 2:** Results From (A) Setups 1–3, Using Pre-Turbulence Centrifugation, and (b) Setup 4, Testing 30 G for 2 Hours and Using Post-Turbulence Centrifugations

a. Results from Setup 1–3															
Analysis	Normal value	RCV (%)	G-Force	Time (Hour)	Control Mean	Control Min	Control Maximum	Control SD	Test Mean	Test Min	Test Maximum	Test SD	N	% Change	P-value Test/ Control
Glucose		2,5	10	2	6,0	5,3	7,3	0,8	5,6	4,5	6,9	0,9	5	−8 %	
			20	2	8,0	4,4	11,2	2,7	6,8	4,1	10,7	2,5	6	−15 %	
			30	1	6,2	4,6	11,0	1,8	5,5	3,7	10,7	1,8	15	−11 %	
				2	7,1	4,9	10,2	2,1	6,4	3,5	9,6	2,1	15	−10 %	
Hemolytic Index		^†^	10	2	6,4	4,0	12,0	3,2	5,0	0	10,0	3,7	5	−22 %	< 0.01
			20	2	5,5	1,0	14,0	4,6	3,2	0	13,0	5,1	6	−42 %	< 0.01
			30	1	5,1	1,0	10,0	3,1	3,1	0	9,0	3,6	15	−39 %	< 0.01
				2	4,0	–	9,0	2,9	2,3	0	10,0	3,5	15	−42 %	< 0.01
Lactate Dehydrogenase	105–205 U/L	25,2	10	2	766	267	1 623	532	882	272	1 821	590	5	15 %	< 0.05
			20	2	350	230	495	103	488	310	679	135	6	39 %	< 0.01
			30	1	221	144	480	92	330	183	584	119	15	49 %	< 0.01
				2	264	128	1 144	251	444	159	1 337	320	15	68 %	< 0.01
Lipid Index		^‡^	10	2	26,4	7,0	88,0	34,5	78,8	29,0	124,0	41,4	5	198 %	< 0.01
			20	2	12,7	7,0	17,0	3,4	65,3	27,0	117,0	28,9	6	416 %	< 0.01
			30	1	15,5	4,0	64,0	14,6	104,8	32,0	339,0	91,6	15	575 %	< 0.01
				2	12,5	4,0	24,0	6,1	88,4	9,0	260,0	79,3	15	609 %	< 0.01
ASAT	15–45 U/L	37,8	30	1	29,5	13,0	75,0	17,8	39,5	18,0	85,0	20,1	15	34 %	< 0.01
				2	24,4	10,0	47,0	10,5	39,2	16,0	72,0	20,7	15	61 %	< 0.01
Folate	>7 nmol/L	72,8	30	1	20,9	7,1	45,0	11,7	24,9	9,8	45,0	13,6	15	19 %	< 0.01
				2	14,8	3,9	26,0	6,7	18,8	4,7	41,0	9,7	15	28 %	< 0.01

b. Results from Setup 4															
Hemolytic Index		^†^	30	2	7,2	6,0	9,0	1,2	5,7	0	9,0	2,8	10	−21 %	< 0.01
Lactate Dehydrogenase	105–205 U/L	25,2	30	2	163	144	185	17	180	156	268	34	10	11 %	ns
Lipid Index		^‡^	30	2	9,0	4,0	13,0	3,6	10,6	6,0	18,0	3,5	10	18 %	ns
ASAT	15–45 U/L	37,8	30	2	18,8	15,0	24,0	3,6	20,7	14,0	29,0	4,4	10	10 %	ns
FOLAT	>7 nmol/L	72,8	30	2	14,6	10,0	18,0	3,2	13,6	8,6	17,0	3,1	10	−7 %	ns

^†^ Footnote: Hemolytic Index >25 influences aspartate aminotransferase, >30 influences lactate dehydrogenase, >50 increases potassium. None of samples had

^‡^ hemolytic indexes >100. J Lipid Index >150 may influence aspartate aminotransferase.

### B. Turbulence and Vibration Simulations

The turbulence and vibration tests were performed in the Environmental Testing Lab of the Norwegian Defense Research Establishment using an LDS V8900 high-force shaker with a frequency range of 5 to 3000 Hz (Brüel & Kjær Sound & Vibration Measurement A/S, DK-2850 Nærum, Denmark). Laser-cut plywood sample racks were constructed to allow for tight, secure fixing of the samples during testing, transport and storage. The sample racks were secured to the shaker table, and sensors were fitted to both the shaker table and the racks (Figure 2).

We found no documented flight data from drones in a relevant configuration for the extreme flying conditions we are exploring; vigorous turbulence and possibly unbalanced propellers due to icing. We aimed to determine the vibration power and frequency range that could provoke changes in blood samples by applying vibrations and shocks to roughly mimic anticipated environments of extreme flying conditions which may be encountered by unspecified drones. The shaker settings were based on measurements from drone flights with a 15-kg DJI Matrice 600 drone, which the Norwegian Defence Research Establishment (FFI) is using in our project. This drone has six two-bladed propellers that rotate at around 2,500–3,000 rpm. Other drones may use alternative configurations, with slower or faster propeller rotation. It is also unknown how drones, combined with payload units for blood sample transport, will cause vibration and shocks in differing speed, wind, and temperatures.

Turbulence was simulated using shocks of 31.75 mm displacement/11 ms from 10–30 G. Initial 2-hour tests conducted at 10 and 20 G showed changes in a few biochemical analytes, but not in hematologic test results; we therefore extended our tests to 30 G for final testing. We acknowledge that this is far higher than most turbulence effects with a rotary wing drone, and even such severe turbulence is likely to be significantly dampened by a heavy rotary wing drone.

**FIGURE 3.**
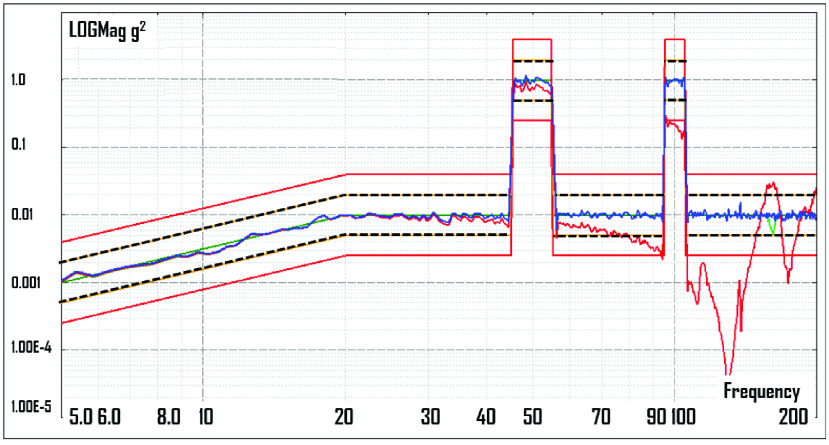
Power spectral density diagram showing the distribution of vibration power over the frequency range of 5 to 200 Hz. The blue (center) line displays the vibrations of the shaker table and the red line displays the sample rack vibrations. The red and the black dotted straight lines are guides used to verify that the forced vibrations are within certain tolerances; in the context of our study, these represent an irrelevant testing laboratory technicality. Both the x and y axes are logarithmic, with the x-axis showing frequency and the y-axis showing g2/Hz. Two desired frequency ranges for significantly higher vibrations are shown, centered at 50 Hz and 100 Hz.

Vibrations were applied in a random distribution of power in the frequency range of 5 to 200 Hz. The root mean square (RMS) value was 4,69G. Spikes of vibration were inserted at 50 ± 5 Hz and at 100 ± 5 Hz, to partially match the vibrations created by propeller rotation and propeller-to-boom interference in a typical drone. The vibrational power spectral density (PSD) diagram, measured on one of the samples themselves, shows considerable damping of vibrations >100 Hz by the plywood sample rack, but below that frequency these racks allow reasonable transfer of the forced spectrum of vibrations to the samples (Figure 3). Future tests could employ a more rigid fixture.

**FIGURE 4.**
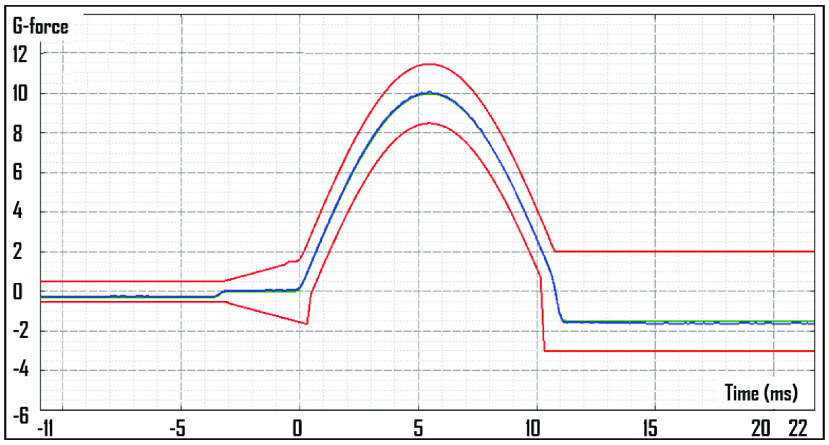
Positive and negative displacement shocks with an amplitude of >30 mm during testing.

We generated 25 turbulence incidents for the 1-hour flights and 50 turbulence incidents for the 2-hour flights. The profile of the shocks representing turbulence is illustrated in Figure 4.

### C. Statistics

We used Bland-Altman analyses to compare turbulence-tested samples with controls. For the analytes that showed changes in response to turbulence and correlated in Pearson correlations, we used multiple stepwise regression to test the correlations. Clinical significance of changes in analytes was assessed analyte-specific by comparing mean differences larger than analytical acceptable difference given by the reference change value (RCV) for each analyte [15].

### D. Ethics

The study was approved by the regional ethics committee for research, Health Authority South-East, Oslo, Norway (reference 2019/182).

## III. Results

### A. Blood Samples

Among the control samples (i.e., before exposure to turbulence), 42.9% of the test results were outside the reference intervals. In the samples for hematologic, biochemistry, and coagulation testing, 49.1%, 39.2%, and 64% of the controls were outside the reference intervals, respectively. All hematologic and most biochemical samples were stable across all test setups and are, for simplicity, presented together in table 1. However, the mean values for aspartate aminotransferase, folate, glucose, lactate dehydrogenase, and lipid index showed substantial changes after turbulence simulation with setups 1–3. The analytes that changed are presented in table 2, showing that changes to some analytes were already evident at 10 G and after only one-hour flights in setup 3. The increase in the test values correlated positively with longer test periods and increasing g-forces.

**FIGURE 5.**
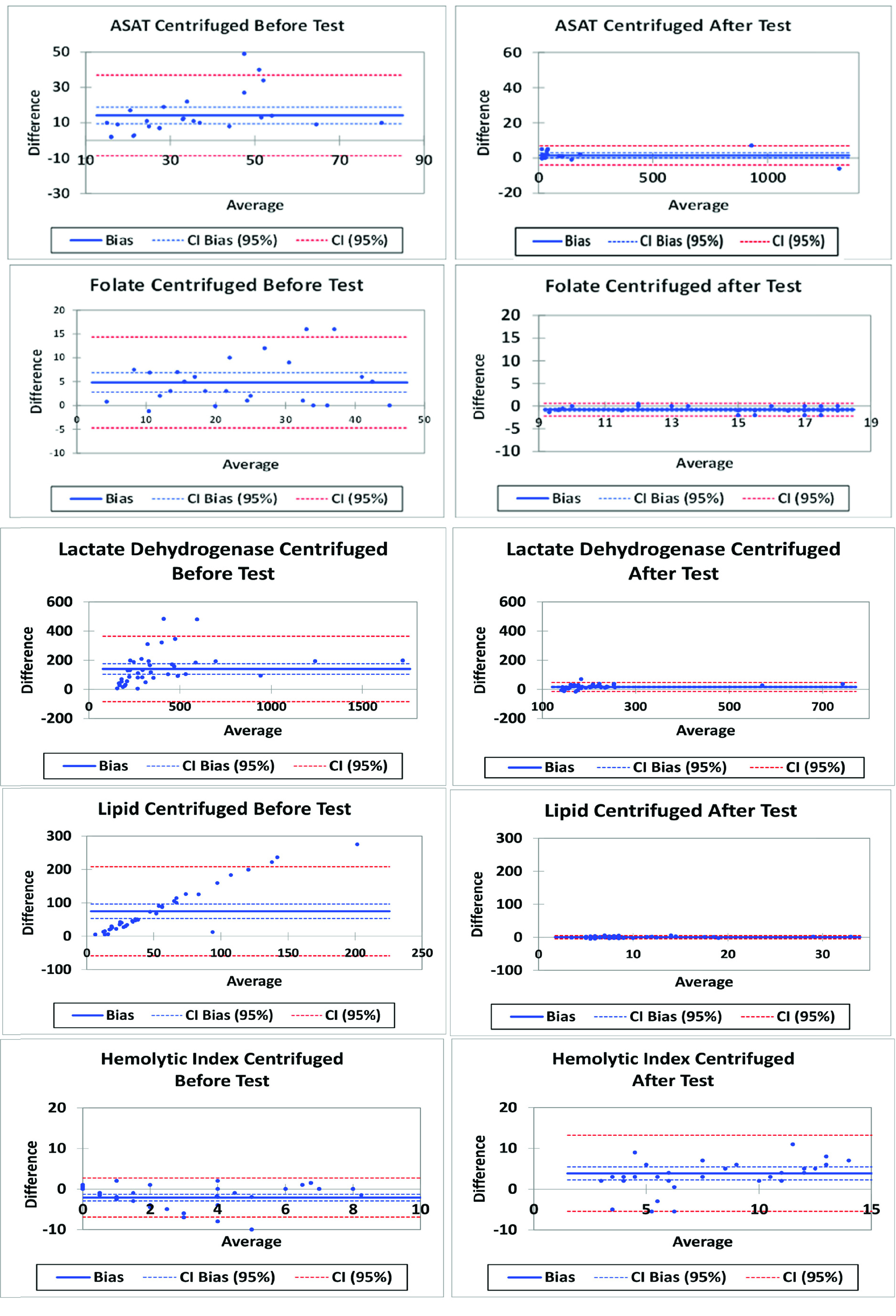
Bland-Altmann plots of the analytes that changed during tests. Effect of centrifugation timing on test results of the analytes influenced by exposure to turbulence. The left panels: centrifugation before exposure to turbulence, the right panels: centrifugation after turbulence.

The Bland Altman analyses from the test results in set up 1–3 are shown in the left panel of Figure 5, illustrating significant deviations between control and test values. In contrast, with setup 4 the changes observed in the same indicators were reduced as shown in the right panel of Figure 5. Whereas samples spun by centrifugation to separate the plasma before turbulence exposure resulted in substantial changes, centrifugation after turbulence eliminated the increases in aspartate aminotransferase, lactate dehydrogenase and lipid index. The decrease in glucose concentration was similar across all setups.

### B. Multivariate Analysis of Factors That May Have Influenced the Test Results

The hemolysis index is an indicator of leakage of hemoglobin from damaged blood cells. With the exception of one patient who scored 122 in the control and 135 in the turbulence test and had to be excluded as the samples could not be analyzed, all other samples had acceptable values and showed decreasing mean hemolysis index values after the test. Among the analytes that we examined, potassium and lactate dehydrogenase are sensitive to hemolysis [16], [17]. In our laboratory, potassium may be elevated with hemolytic values above 50, and potassium analysis is not accepted when hemolytic values exceed 100. Lactate dehydrogenase may be elevated at lower hemolytic values (above 30 in our laboratory), and is also not reported if hemolytic values exceed 100. Hemolytic index values in this study were all below these levels.

Based on the Pearson correlations, we performed multivariate correlations between the analytes that changed in response to turbulence, including the hemolysis index (Table 3). There was no correlation between the hemolysis index and changes in lipid index, nor did the hemolytic index correlate with the other tests in multivariate analysis when the lipid index was included. For all variables, the best regression model included both centrifugation and changes in lipid index, which eliminated the statistical significance of centrifugation.

**TABLE 3:** Multivariate Analysis of Analytes That Changed in Response to Turbulence Exposure and Had Significant Pearson Correlations in Univariate Analysis

Variable		R	R2	Adjusted R2	Stell. Error of the Estimate	Unstandardized Coefficient		Sid. Error	Standardized Coefficient			t	Sig.
Lipid index change	Constant	0.77	0.60	0.59	2.12		0.52	0.57		–		0.92	0.35
	Time						0.29	0.37	–	0.05	–	0.78	0.44
	Centrifugated						5.27	0.45		0.79		11.79	0.00
	Constant	0.78	0.61	0.60	2.11		0.66	0.57		–		1.13	0.25
	Time						0.27	0.37	–	0.05	–	0.72	0.47
	Centrifugated						4.95	0.49		0.74		10.10	0.00
	Change Hemolytic Index					–	0.00	0.05	–	0.11	–	1.58	0.12
Aspartate amingtransferase change	Constant	0.59	0.35	0.33	7.96		1.14	2.42				0.47	0.64
	Time						1.81	1.60		0.12		1.13	0.26
	Centrifugated						10.86	1.99		0.56		5.45	0.00
	Constant	0.59	0.35	0.32	8.03	–	1.12	2.44		–	–	0.46	0.65
	Time						1.81	1.61		0.12		1.12	0.27
	Centrifugated						10.79	2.07		0.56		5.22	0.00
	Change Hemolytic Index					–	0.03	0.23	–	0.02	–	0.14	0.89
	Constant	0.72	0.52	0.49	6.93	–	2.01	2.11		–	–	0.95	0.35
	Time						2.12	1.30		0.14		1.52	0.13
	Centrifugated						1.14	2.72		0.06		0.42	0.6S
	Change Lipid index						1.78	0.38		0.64		4.64	0.00
LD change	Constant	0.59	0.35	0.33	7.96	–	1.14	2.42		–	–	0.47	0.64
	Time						1.81	1.60		0.12		1.13	0.25
	Centrifugated						10.86	1.99		0.56		5.45	0.00
	Constant	0.54	0.29	0.27	0.39		–	0.10		–		0.02	0.98
	Time						0.05	0.07		0.07		0.79	0.43
	Centrifugated						0.51	0.09		0.56		5.71	0.00
	Change Hemolytic Index						0.01	0.01		0.15		1.54	0.13
	Constant	0.72	0.52	0.49	6.93	–	2.01	2.11		–	–	0.95	0.34
	Time						2.12	1.39		0.14		1.52	0.13
	Centrifugated						1.14	2.72		0.06		0.42	0.68
	Change Lipid index						1.78	0.38		0.64		4.64	0.00
Folate change	Constant	0.58	0.34	0.32	0.27	–	0.17	0.08		–	–	2.24	0.03
	Time						0.03	0.05		0.06		0.66	0.51
	Centrifugated						0.39	0.06		0.57		6.19	0.00
	Constant	0.59	0.35	0.32	0.27	–	0.15	0.08		–	–	1.98	0.05
	Time						0.03	0.05		0.06		0.64	0.53
	Centrifugated						0.36	0.07		0.52		5.30	0.00
	Change Hemolytic index					–	0.01	0.01	–	0.12	–	1.29	0.20
	Constant	0.65	0.42	0.40	0.26	–	0.18	0.07		–	–	2.59	0.01
	Time						0.04	0.05		0.07		0.33	0.41
	Centrifugated						0.12	0.10		0.17		119	0.24
	Change Lipid Index.						0.05	0.01		0.49		3.49	0.00

## IV. Discussion

This study demonstrated that most of the blood samples were resistant to a combination of random vibration and multiple episodes of turbulence at 10–30 G, the most extensive being two hours with 30 G. Interestingly, the samples with separation gel that were not separated by centrifugation prior to being exposed to vibration and turbulence seemed more resistant to vibration and turbulence than the samples in tubes containing a plasma separator gel and spun before turbulence testing. Our interpretation is that the separation of plasma for biochemical analysis should be performed after drone transportation.

Those analytes that resisted turbulence did so across all levels of turbulence, whereas those that changed after turbulence test did so already at the first G-level we tested (10 G) and showed an increase in changes with further increase to the 30 g-level and also from 1 to two hours of test flight.

Although patients were anonymous, the blood specimens were taken from clinics where patients have diseases ranging from cancer, leukemia, and severe anemia, to immunopathological disorders, the treatments of which could have varying effects on patient physiology. This is reflected by the values of blood analytes, which varied from severely low to highly pathologic. This allowed us to study a previously unexplored topic: the effects of drone transport turbulence on clinical samples far outside the normal range.

In the patient groups we studied, the effect of turbulence on blood analytes was similar to that of normal samples. These findings address an outstanding issue discussed in the report on drone flights by Amukele *et al.* [7].

Pre-analytic centrifugation of gel tubes is routinely performed and has been validated as not causing significant changes in analytes even at relative centrifugal forces (RCFs) of 2,$000\times $ g and above [11], [18]. A previous study by Møller *et al.* [12], who tested a shortened centrifugation time with an RCF of 3,$000\times $ g, concluded that these conditions were safe for blood sample analytes with the exception of lactate dehydrogenase, which showed a higher-than-acceptable coefficient of variation. Another study reported significant changes in the lipid index [13], and both of these studies reported observations similar to ours. The effects of g-forces have also been reported in studies validating pneumatic tube systems (PTS). Both Farnsworth *et al.* and Mullins *et al.* [10], [19] reported clinically significant increases in lactate dehydrogenase concentration. Mullins *et al.* found that changes in lactate dehydrogenase concentration and hemolytic index was proportional to the elapsed transport time and number of PTS-associated shocks. Streichert *et al.* [17], using vibration and turbulence, reported similar relationships between PTS acceleration speed and changes in aspartate aminotransferase and lactate dehydrogenase as we observed. By contrast, Gils *et al.* [14] concluded that there was no effect of PTS transport with peak g-forces of up to 15 G.

Of note, a recent review of the literature related to the PTS [20] was unable to supply evidence for the safety of PTS use in blood sample transportation due to the high degree of study heterogeneity.

G-forces triggered by centrifugation may be more static than physical forces related to the PTS or drone turbulence. The physical strain on sample tubes that we generated by simulating vibration and turbulence may effectively mimic the conditions of PTS transport, but might differ from the g-forces caused by shaking during centrifugation.

Our observations regarding the impact of centrifugation of gel tubes before or after exposure to turbulence may be interpreted in several ways. During centrifugation of tubes containing a separator gel, the gel gradually moves upward from the bottom of the tube to form a barrier between the serum and blood cells. In this process, blood cells are transported through the gel, making it more porous and probably more prone to disintegration during vibration and turbulence. Separator gels from different manufacturers might have different chemical formulas, and thus different levels of resistance to g-forces. Accordingly, the g-force required to trigger upward movement of the gel may vary depending on the tube brand. Gel that remains at the bottom of the tube before centrifugation may be less prone to damage than gel after transformation during centrifugation and then located higher in the tube. It is possible that the turbulence applied in our 1- and 2-hour simulations exceeded a tolerance level that is not challenged during the 5–20 minutes of standard centrifugation in the laboratory setting, even when higher g-forces are used for centrifugation. Cell damage could also be suspected, but then an increase in potassium would have been expected.

A relevant topic for drone transport of biologic materials is temperature, which may influence changes in blood analytes over time, including glucose, potassium and several enzymes [21]–[23]. Currently, most biologic samples acquired in clinical settings require cooling or special temperatures during storage and transport to slow down the biologic processes. Alterations that occur in such samples within the first 4–8 hours tend to be small, but substantial changes may occur after 16–24 hours, and have more of an impact on plasma than serum [22], [23]. Active temperature regulation during drone transport will require energy, the generation of which may cause increased drone weight. To optimize drone transport complexity with respect to such conditions, time intervals for transport must be short enough to minimize the demand for specific transport temperatures. Logistic models that ensure transport with minimal delays will therefore be crucial, and cohorts that meet such conditions must be further studied. We maintained all of the analytes at room temperature (range, 18-22°C) to create a study model that was as simplistic as possible. Future transport containers should be able to maintain a fixed inner temperature over time regardless of the outer temperature, and preferably without energy consumption.

All our test samples were treated identically except for the turbulence simulation. The control and test samples were analyzed at the same time in the afternoon and the set-ups were performed within similar time spans. Furthermore, we found no changes in plasma analytes that were not subjected to centrifugation before exposure to vibration and turbulence. Under the current standard in our laboratory, plasma samples should be separated by centrifugation within 2 hours of collection. Separation of plasma by centrifugation before drone transport would represent a critical obstacle for the development of time-intensive drone transport solutions. We propose that altering the standard procedure to allow for “flying” of plasma gel sample tubes prior to centrifugation should be the preferred routine for drone transport. By optimizing the logistics, we expect to successfully transport unseparated plasma samples to the analyzing laboratory by drones, well within the required time window. Flight times longer than 2 hours may require further attention to this topic.

A primary intention for this project was to study biologic characteristics that could be used to determine drone quality specifications which must be satisfied for drones to be licensed for biologic transport. A relevant question is whether flight duration >2 hours and turbulence conditions >30 G will have to be tested for future drone services. We believe that a g-force of 30 G is unlikely to be experienced in most drone applications and therefore should represent an upper limit of conceivable g-force due to extreme turbulence. It is unknown whether any RW drones have recorded the effects of such turbulence, but this upper limit represents extremely violent turbulence that would be very unlikely to impact on the cargo of RW drones, which have a significant damping effect in flight. Most RW drones can be expected to experience only single-digit g-forces, whereas some fixed wing air vehicles may behave quite differently, with potentially higher g-forces during turbulence or strong wind shear situations.

We have shown that changes in centrifuged samples may occur with 10 G and seem to increase with increasing turbulence and exposure time. Whether the samples centrifuged that were resistant to turbulence when centrifuged after test will still be stable beyond two hours, simulating longer flight times, is not known and should be further studied.

Effects of the combination of vibration and turbulence on drone transport are not easy to study from drone flights alone. Wind conditions may be unpredictable and vary across different ground elevations, buildings and so on, and establishing a wide enough span of relevant physical data would be quite laborious under such conditions. In vitro simulations represent an alternative to real flights, and offer controlled conditions. Future studies should include a range of temperatures and atmospheric pressures. Although we expect that our simulation of 30 G turbulence goes sufficiently beyond the conditions that drones routinely may encounter in the future, the combination of temperature, oxygen levels and humidity may also be of importance.

### A. Strengths and Limitations

We have simulated the effects of vibration and turbulence on blood samples representing a large variety of pathologies and with baseline values considerably outside normal limits. We believe that our results reflect the conditions of everyday clinical populations in a large hospital and thus have general applicability. The usefulness for other analyses, such as blood gas, hormones and specific proteins will require further studies.

We cannot exclude the possibility that blood from patients with complex diseases who may be using steroids, cytostatic or immunosuppressive medications may have special needs that impose specific technical drone transport solutions. It may represent a limitation in our study that different tolerance of analytes in such specific patient groups may have been concealed in our data [10]. Furthermore, counting of cells does not cover function testing, for example in platelets. The detailed exploration of such conditions may be a rather demanding task, and defining specific criteria of drone transport for samples from special patient groups would probably not be an efficient approach.

Our study focused on blood samples. Successful drone transport of other types of specimens, such as body fluids, microbiologic samples and tissue samples, may require special attention or specific settings that may limit potential transport time. Accordingly, we need further research on these topics; in particular, how to establish drone transport logistics with short transport times.

As we found no actual flight data from drones in a relevant configuration for the extreme flying conditions we are exploring; vigorous turbulence and possibly unbalanced propellers due to icing, we had no a priori data to choose the physical environment we simulated. We investigated vibration power and frequency range in these first exploratory tests as a starting point to provoke changes in blood samples, applying vibrations and shocks to roughly mimic anticipated environments of extreme flying conditions which may be encountered by unspecified drones. However, as vibration frequencies will vary across differing drone brands, sizes, and velocities, the frequency and turbulence simulations we used are not universally applicable to all types of drone transport.

Differing altitudes may cause variations in air pressure and oxygen. However, with the limited height anticipated for the future civil drone space, we expect that such perspectives will be covered by transport compartment designs that ensure constant physical conditions at prespecified values.

## V. Conclusion

Blood samples from patients with a wide range of diseases can tolerate substantial vibration and turbulence lasting up to 2 hours. Test tubes containing a separator gel may generate changes in test results if subjected to centrifugation before transportation. Such changes were avoided by delaying centrifugation until after the exposure to turbulence. Drone transport under heavy air turbulence is likely to become a realistic service applicable to a large variety of medical needs, but more research on quality assessment is needed to ensure the future of drone transport in health care.

## Appendix 1

See Table 4.

**TABLE 4:** Overview of Methods and Reagents Used for Analyses

Analysis	Principle	Reagent
Hematology	SysmexXN-9000	
White blood cells, Basophilic granulocytes, Eosinophilic granulocytes, Monocytes, Lymphocytes, Neutrophilic granulocytes	Optical fluorescence	Lysercell WNR, Fluorocell WNR
Hemoglobin	Photometry of sodium lauryl sulphate binding to hemoqlobin	Sulfolyser
Hematocrit	Estimate based on red blood cell count	
Red blood cell count	Measurement of Impedance	
Reticulocytes	Flow cytometry	Fluorocell (RET), Cellpack DFL
Mean corpuscular hemoglobin	Estimate based on hemoglobin and red blood cell count	
Mean corpuscular hemoglobin concentration	Estimate based on hemoglobin and hematocrit	
Mean corpuscular volume	Estimate based on hematocrit and red blood cell count	
Platelet count	Measurement of Impedance	Cellpack DCL/DCT
Mean platelet volume	Estimate based on platelet volume and platelet count	
Biochemistry	Roche Cobas 8000	
ALAT	Pyridoxal phosphate activation and measurement of absorbance reduction	ALTPL (Roche)
Calcium	Colorimetry of calcium and NM-BAPTA complex	CA2 (Roche)
Chloride	Ion selective electrode (Indirect)	Chloride electrode
CRP	Particle enhanced immunoturbidimetry	CRPL3 (Roche)
Magnesium	Colorimetry	MG2 (Roche)
Potassium, Sodium	Ion selective electrode (Indirect)	Potassium/Sodium electrode
Triglyceride	Enzymatic colorimetry	TRIGL (Roche)
Glucose	Enzymatic (hexokinase)	GLUC3 (Roche)
Lactate dehydrogenase	Enzymatic	LDHI2 (Roche)
ASAT	Pyridoxal phosphate activation and	ASTLP (Roche)
	measurement of absorbance reduction	
Folic acid	Electrochemoluminescence immunoassay	Elecsys folate III (Roche)
Hemolytic Index, Lipid index	Absorptiometry	
Coagulation	Stago StaR	
Thromboplastin time	Plasma clotting test	STA-PPT Automate 5 (Stago)
D-dimer	Immunoturbidimetry	STA-LIATEST D-DI PLUS (Stago)
Fibrinogen	Plasma clotting test	STA-Liquid Fib STA (Stago)
INR	Plasma clotting test	Diagnostica Stago SPA+ (Stago)
